# Comparison of Physical and Mechanical Properties of Various Commercially Available Nanohybrid Composites: An In Vitro Study

**DOI:** 10.7759/cureus.113203

**Published:** 2026-07-23

**Authors:** Manmeet Kaur, Rajinder Bansal, Manu Bansal, Mamta Singla, Sakshi Singla, Romil Arora

**Affiliations:** 1 Department of Conservative Dentistry and Endodontics, Guru Nanak Dev Dental College and Research Institute, Sunam, IND; 2 Department of Conservative Dentistry and Endodontics, Faculty of Dental Sciences, Shree Guru Gobind Singh Tricentenary University, Gurugram, IND

**Keywords:** composite resins, dental materials, flexural strength, hardness, mechanical properties

## Abstract

Introduction: Nanohybrid resin composites are widely used in restorative dentistry because they combine favorable esthetics and improved mechanical properties. However, commercially available nanohybrid composites differ in their filler characteristics, resin matrix composition, and manufacturing processes, which may influence their clinical performance. Therefore, this study aims to compare the tensile strength, flexural strength, compressive strength, and Barcol hardness of four commercially available nanohybrid composite resins.

Materials and methods: This in vitro experimental study was conducted using 160 standardized specimens prepared from four types of nanohybrid composite resins: Tetric N-Ceram, Fusion Universal, NanoCom Composite, and NanoFill Composite. Each material was allocated to 40 specimens, which were further divided into four subgroups (n = 10) for tensile, flexural, compressive, and Barcol hardness testing. Bar-shaped specimens were fabricated for tensile and flexural strength evaluation, whereas cylindrical specimens were prepared for compressive strength and hardness testing. Mechanical testing was performed using a universal testing machine and Barcol hardness tester under standardized laboratory conditions. Data were analyzed using one-way analysis of variance followed by Tukey's post-hoc test, with statistical significance set at p < 0.05.

Results: Tetric N-Ceram demonstrated the highest mean tensile strength (22.52 ± 3.79 MPa), flexural strength (94.36 ± 21.65 MPa), compressive strength (87.82 ± 55.60 MPa), and Barcol hardness (73.60 ± 2.32). Significant intergroup differences were observed in the tensile strength (p = 0.038), flexural strength (p = 0.002), and Barcol hardness (p < 0.001), whereas the compressive strength was not significantly different among the four materials (p = 0.555). Pairwise comparisons demonstrated significantly greater tensile and flexural strengths for Tetric N-Ceram compared with several other composites, while the Barcol hardness also differed significantly between most material combinations.

Conclusion: The mechanical performance of commercially available nanohybrid composite resins differed significantly. Tetric N-Ceram demonstrated the most favorable overall mechanical properties, particularly with respect to tensile strength, flexural strength, and surface hardness. These findings indicate that nanohybrid composites are not mechanically equivalent and should be selected based on scientific evidence rather than on material classification alone.

## Introduction

Resin-based composite materials have become the restorative material of choice in modern dentistry because of their excellent esthetic properties, compatibility with adhesive bonding techniques that enable durable attachment to tooth structure, and support for minimally invasive cavity preparation [1]. Continuous advancements in composite technology have focused on improving the mechanical performance and longevity of restorations, while maintaining superior esthetics. These developments have led to modifications in resin matrix composition, filler characteristics, and polymerization systems, resulting in materials with enhanced clinical performance [2].

Among the various generations of resin composites, nanohybrid composites represent significant advancements in restorative dentistry. These materials combine nanosized filler particles with conventional microfillers, enabling a higher filler loading, improved polish retention, reduced polymerization shrinkage, and enhanced wear resistance. The incorporation of nanotechnology has also contributed to better mechanical properties and color stability by improving the stress distribution within the resin matrix and strengthening the filler-matrix interface. Consequently, nanohybrid composites are widely recommended for both anterior and posterior restorations, where esthetics and mechanical durability are equally important [3,4]. Although these restorative materials are collectively classified as nanohybrid composites, they are not compositionally identical. Commercially available nanohybrid composites differ in filler type, filler particle size and distribution, filler loading (by weight and volume), resin matrix composition, silane coupling chemistry, photoinitiator systems, and manufacturing processes. These variations may substantially influence their physical and mechanical properties, indicating that materials within the same classification should not be assumed to exhibit equivalent clinical or laboratory performance.

The long-term clinical success of composite restorations largely depends on their physical and mechanical properties. Tensile strength, flexural strength, compressive strength, and surface hardness are critical indicators of a material's ability to withstand functional occlusal forces, resist fractures, and maintain dimensional stability during service [5]. However, despite belonging to the same material category, commercially available nanohybrid composites differ considerably in terms of filler composition, particle size, filler loading, resin chemistry, and manufacturing processes [6]. These variations may result in significant differences in mechanical behavior, making evidence-based comparisons essential for appropriate material selection by clinicians.

Therefore, the present in vitro study was conducted to compare the physical and mechanical properties of four commercially available nanohybrid composite resins. This study aimed to evaluate and compare the tensile strength, flexural strength, compressive strength, and Barcol hardness of Ivoclar Tetric N-Ceram, Prevest Fusion Universal, Dentgist NanoCom, and Waldent NanoFill composites. The objectives were to measure each mechanical property using standardized laboratory testing methods, compare the performances of the four materials, and identify the composite exhibiting the most favorable overall mechanical characteristics for clinical restorative applications.

## Materials and methods

Study design, setting, and duration

This in vitro study was conducted in the Department of Conservative Dentistry and Endodontics, Guru Nanak Dev Dental College and Research Institute, Sunam, Punjab, India, over a period of seven months from June 2025 to December 2025. As the study involved only commercially available restorative materials and did not include human participants, extracted teeth, patient records, animal tissues, or biological specimens, institutional ethical approval was not required.

Sample size estimation

The sample size was calculated using G*Power software (version 3.1.9; Heinrich Heine University Düsseldorf, Düsseldorf, Germany). A priori power analysis for one-way analysis of variance (ANOVA) was performed using an effect size (f) of 0.28, which was derived from a previous in vitro investigation [7], with a statistical power of 80% and a two-sided significance level of 5%. The minimum sample size required was estimated to be 145. To compensate for possible specimen loss during fabrication or testing, the sample size was increased by approximately 10%, resulting in a final sample size of 160 specimens. These included 80 bar-shaped and 80 cylindrical specimens, with 40 specimens allocated to each composite group. Each group was further subdivided into four subgroups (n = 10) for tensile strength, flexural strength, compressive strength, and Barcol hardness tests.

Study materials and group allocation

**Figure 1:**
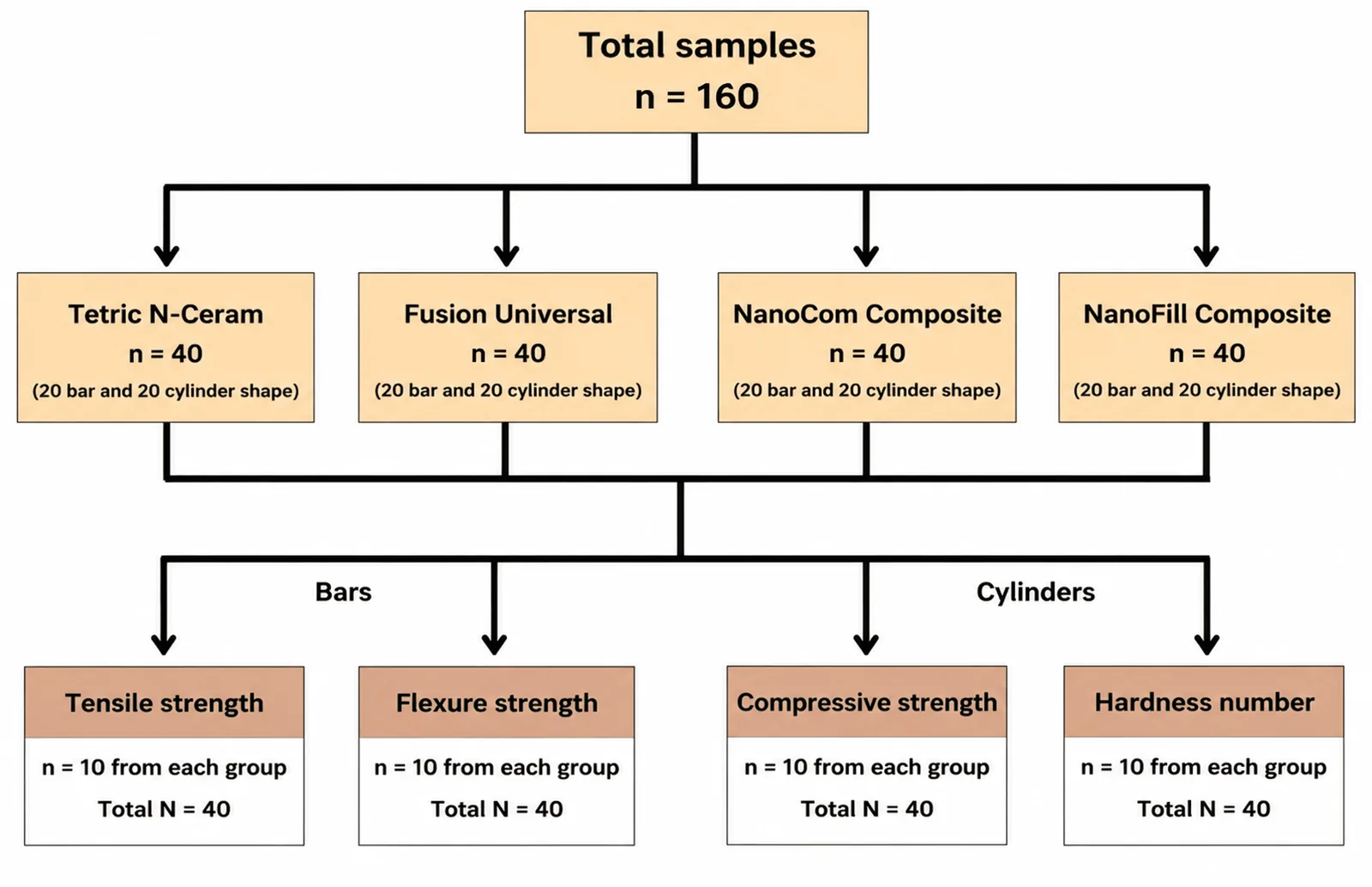
Group allocation of study samples. Image created with Canva Pty Ltd (Surry Hills, Sydney, Australia).

A total of 160 specimens were prepared and allocated equally into four experimental groups (n = 40 each) according to the nanohybrid composite resin evaluated: Group I, Tetric N-Ceram (Ivoclar Vivadent AG, Schaan, Liechtenstein); Group II, Fusion Universal (Prevest DenPro Limited, Jammu, India); Group III, NanoCom Composite (DentGist, New Delhi, India), and Group IV, NanoFill Composite (Waldent Innovations Pvt. Ltd., New Delhi, India). Each group was comprised of 20 bar-shaped specimens (25 mm × 2 mm × 2 mm) and 20 cylindrical specimens (5 mm × 10 mm). The bar-shaped specimens were further subdivided into two subgroups (n = 10 each) for tensile strength and flexural strength testing, whereas the cylindrical specimens were similarly divided into two subgroups (n = 10 each) for compressive strength and Barcol hardness evaluation. Thus, each composite group consisted of four subgroups of ten specimens, corresponding to the four mechanical tests. All restorative materials were procured from authorized distributors, stored according to the manufacturers' recommendations, and used before their expiry date. Specimen fabrication and polymerization were performed in strict accordance with the manufacturer’s instructions to ensure uniformity and minimize experimental variability (Figure 1).

Operator calibration and standardization

To minimize procedural variability, all specimens were fabricated by a single trained investigator who underwent calibration before the commencement of the study. A pilot standardization exercise involving 20 trial specimens was performed to ensure the reproducibility of specimen fabrication, incremental placement, light-curing protocol, finishing procedures, and testing methodology. The dimensions of all specimens were verified using a digital Vernier caliper with an accuracy of 0.01 mm, and only specimens within ±0.1 mm of the prescribed dimensions were accepted. The same visible light-emitting diode (LED) curing unit (Woodpecker Mini S LED Curing Light, Guilin Woodpecker Medical Instrument Co., Ltd., Guilin, China) operating at an output intensity of approximately 1000 mW/cm² was used throughout the study. The curing light intensity was periodically verified using a radiometer before specimen preparation each day. Mechanical testing was performed by the same operator using identical testing parameters for all the specimens to eliminate inter-operator variability.

Specimen preparation

A total of 160 specimens were fabricated under standardized laboratory conditions. Eighty bar-shaped specimens (25 mm × 2 mm × 2 mm) were prepared for tensile and flexural strength testing using customized addition silicone molds fabricated from addition silicone putty (Tech-Sil® S25 Addition Silicone Putty, Techno-Sil, Bidford-on-Avon, United Kingdom). Equal proportions of the base and catalyst were hand-mixed until a homogeneous consistency was obtained, following which a stainless-steel template of the desired dimensions was embedded into the unset putty to create a standardized mold. After complete polymerization of the putty, the molds were used for specimen fabrication.

The composite resin was incrementally packed into the molds in horizontal layers. A transparent polyester Mylar strip was placed over the composite surface to obtain a smooth, oxygen-inhibited-free surface and remove excess material. Each 2-mm increment was polymerized using a visible LED curing unit operated in the standard continuous curing mode at an output intensity of approximately 1000 mW/cm² for 20 seconds, in accordance with the manufacturer's recommendations. During curing, the light guide tip was positioned perpendicular to and in direct contact with the Mylar strip covering the specimen surface.

After specimen fabrication and 24-hour storage in distilled water, all specimens were visually inspected by a single calibrated investigator under standardized illumination for surface defects, including voids, air bubbles, cracks, incomplete polymerization, and surface irregularities. Specimen dimensions were verified using a digital Vernier caliper with an accuracy of 0.01 mm. Only specimens exhibiting complete polymerization, smooth surfaces, uniform dimensions (within ±0.1 mm of the prescribed dimensions), and absence of visible defects were included in the study. Specimens demonstrating dimensional inaccuracies, voids, air bubbles, cracks, incomplete curing, surface irregularities, or fractures during fabrication or storage were excluded and replaced with newly fabricated specimens.

For the evaluation of compressive strength and Barcol hardness, 80 cylindrical specimens measuring 5 mm in diameter and 10 mm in height were fabricated using standardized plastic cylindrical molds. The composite resin was inserted in 2-mm increments, with each increment polymerized for 20s. Following the placement of the final increment, an additional Mylar strip was applied over the surface before final light curing. After complete polymerization, the plastic molds were longitudinally sectioned using a Bard-Parker blade, and the specimens were carefully retrieved. All the fabricated specimens were stored individually in distilled water at 37°C for 24 h before mechanical testing to simulate oral conditions and ensure uniform post-polymerization.

Mechanical testing

Direct tensile strength (uniaxial tensile strength) was evaluated using a Universal Testing Machine (Instron® Universal Testing Machine, Instron Corporation, Norwood, Massachusetts, USA). Bar-shaped specimens were secured in the testing grips, and a uniaxial tensile load was applied at a crosshead speed of 1 mm/min until fracture. The maximum load at failure was recorded, and tensile strength was calculated and expressed in megapascals (MPa).

The flexural strength was determined by a three-point bending test using the same Universal Testing Machine. Each specimen was positioned on two supporting rollers, and the load was centrally applied at a crosshead speed of 3 mm/min until fracture. Flexural strength was calculated and recorded in MPa.

Compressive strength testing was performed using cylindrical specimens positioned centrally between the compression plates of the Universal Testing Machine. Compression was applied at a constant crosshead speed of 1 mm/min until specimen failure, and the compressive strength values were expressed in MPa.

The surface hardness was assessed using a Barcol Impressor (Model GYZJ-934-1 Barcol Hardness Tester, Barber-Colman Company, Rockford, Illinois, USA) following ASTM D2583 specifications. A diamond indenter was applied perpendicular to the specimen surface under a standardized load, and the indentation reading was recorded after 2 s to obtain the Barcol hardness number.

Outcome measures

The primary outcome variables included the mean tensile strength (MPa), flexural strength (MPa), compressive strength (MPa), and Barcol hardness of each nanohybrid composite resin. These parameters were compared to determine the differences in the physical and mechanical performance of the tested restorative materials.

Statistical analysis

Data were entered into Microsoft Excel (Microsoft Corporation, Redmond, Washington, USA) and analyzed using IBM SPSS Statistics for Windows, Version 25 (Released 2017; IBM Corp., Armonk, New York, USA). Data normality was assessed using the Shapiro-Wilk test. Continuous variables are expressed as mean ± standard deviation and 95% confidence intervals. Intergroup comparisons were performed using one-way ANOVA followed by Tukey's honest significant difference (HSD) post-hoc test for pairwise comparisons. Statistical significance was set at p < 0.05.

## Results

The descriptive statistics of the evaluated mechanical properties are presented in Table 1. Tetric N-Ceram (Group I) demonstrated the highest mean tensile strength (22.52 ± 3.79 MPa), flexural strength (94.36 ± 21.65 MPa), compressive strength (87.82 ± 55.60 MPa), and Barcol hardness (73.60 ± 2.32 MPa). Waldent NanoFill (Group IV) exhibited the lowest mean tensile strength (14.08 ± 6.20 MPa) and compressive strength (64.40 ± 45.69 MPa), whereas DentGist NanoCom (Group III) demonstrated the lowest flexural strength (57.48 ± 16.75 MPa) and Barcol hardness (56.00 ± 2.83). The 95% confidence intervals for each outcome measure are presented in Table 1.

**Table 1:** Descriptive analysis of tensile strength, flexural strength, compressive strength, and hardness among the four nanohybrid composite resins. Data are presented as mean ± standard deviation and 95% confidence interval. CI = confidence interval; SD = standard deviation; MPa = megapascal.

Property	Group I (Tetric N-Ceram)		Group II (Fusion Universal)		Group III (Dentgist NanoCom)		Group IV (Waldent NanoFill)	
	Mean ± SD	95% CI of Mean	Mean ± SD	95% CI of Mean	Mean ± SD	95% CI of Mean	Mean ± SD	95% CI of Mean
Tensile strength (MPa)	22.52 ± 3.79	19.81 – 25.23	20.10 ± 4.30	17.02 – 23.18	21.65 ± 10.74	13.97 – 29.33	14.08 ± 6.20	9.65 – 18.51
Flexural strength (MPa)	94.36 ± 21.65	78.87 – 109.85	80.20 ± 13.73	70.38 – 90.02	57.48 ± 16.75	45.49 – 69.47	60.12 ± 9.02	53.67– 66.57
Compressive strength (MPa)	87.82 ± 55.60	48.05 – 127.59	71.48 ± 32.08	48.54 – 94.43	84.31 ± 24.20	67.00 – 101.62	64.40 ± 45.69	31.72 – 97.09
Barcol hardness number	73.60 ± 2.32	71.91 – 75.26	66.00 ± 3.56	63.45 – 68.55	56.00 ± 2.83	53.98 – 58.02	72.60 ± 3.44	70.14 – 75.06

Intergroup comparisons using one-way ANOVA revealed statistically significant differences in tensile strength (p = 0.038), flexural strength (p = 0.002), and Barcol hardness (p < 0.001), whereas compressive strength did not differ significantly between the groups (p = 0.555) (Table 2). The largest effect size was observed for Barcol hardness (0.41), followed by flexural strength (0.28) and tensile strength (0.23), whereas compressive strength demonstrated only a negligible effect size (0.04).

**Table 2:** Intergroup comparison of tensile strength, flexural strength, compressive strength, and Barcol hardness among the four nanohybrid composite resins. Statistical analysis was performed using one-way analysis of variance (ANOVA), η² = eta squared effect size, and p < 0.05 was considered statistically significant; * indicates a statistically significant difference.

Property	Intergroup comparison		
	F value	p-value	Effect size (η²)
Tensile strength	3.10	0.038*	0.23
Flexural strength	6.12	0.002*	0.28
Compressive strength	0.71	0.555	0.04
Barcol hardness number	64.21	< 0.001*	0.41

Post-hoc pairwise comparisons performed using Tukey's test revealed distinct statistical significance patterns for tensile and flexural strengths across the four nanohybrid composite resins. Regarding tensile strength, Waldent NanoFill (Group IV) exhibited significantly lower values compared to Tetric N-Ceram (p = 0.006), Fusion Universal (p = 0.034), and DentGist NanoCom (p = 0.019). Conversely, tensile comparisons among Groups I, II, and III were non-significant. For flexural strength, Tetric N-Ceram significantly outperformed DentGist NanoCom and Waldent NanoFill, with both comparisons showing p < 0.001. Additionally, Fusion Universal demonstrated significantly greater flexural strength than DentGist NanoCom (p = 0.007) and Waldent NanoFill (p = 0.010). In contrast, the flexural strength between Tetric N-Ceram and Fusion Universal (p = 0.119) and between DentGist NanoCom and Waldent NanoFill (p = 0.962) did not reach statistical significance (Table 3).

**Table 3:** Pairwise comparison of tensile strength and flexural strength among the four nanohybrid composite resins. Statistical analysis was performed using Tukey's honest significant difference (HSD) post-hoc test following one-way analysis of variance. Data are presented as mean differences with 95% confidence intervals. Group I = Tetric N-Ceram, Group II = Prevest Fusion Universal, Group III = Dentgist NanoCom, Group IV = Waldent NanoFill. p < 0.05 was considered statistically significant; * indicates a statistically significant difference. CI = confidence interval; MPa = megapascal.

Intergroup comparison	Tensile strength				Flexural strength			
	Mean difference (MPa)	95% CI (Lower–Upper)	t-value	p-value	Mean difference (MPa)	95% CI (Lower–Upper)	t-value	p-value
Group I vs. Group II	2.42	−1.90 to 6.74	2.67	0.442	14.16	−2.74 to 31.06	3.49	0.119
Group I vs. Group III	0.87	−5.52 to 7.26	0.48	0.969	36.88	19.02 to 54.74	8.51	<0.001*
Group I vs. Group IV	8.44	2.78 to 14.10	7.34	0.006*	34.24	18.56 to 49.92	9.23	< 0.001*
Group II vs. Group III	-1.55	−7.94 to 4.84	–0.84	0.914	22.72	5.82 to 39.62	6.63	0.007*
Group II vs. Group IV	6.02	0.36 to 11.68	5.04	0.034*	20.08	4.40 to 35.76	7.72	0.010*
Group III vs. Group IV	7.57	1.18 to 13.96	3.85	0.019*	-2.64	−18.32 to 13.04	–0.87	0.962

The post-hoc comparisons of the compressive strength and Barcol hardness are summarized in Table 4. No statistically significant differences were observed in compressive strength among any of the four composite groups following the Bonferroni correction (all p > 0.05). In contrast, barcol hardness differed significantly between most pairwise comparisons. Tetric N-Ceram demonstrated significantly higher hardness values than Fusion Universal and DentGist NanoCom (both p < 0.001), and Fusion Universal also exhibited significantly greater hardness than DentGist NanoCom (p < 0.001). Waldent NanoFill showed hardness values comparable to Tetric N-Ceram (p = 0.768), but significantly higher than that of DentGist NanoCom (p < 0.001) and significantly lower than that of Fusion Universal (p < 0.001).

**Table 4:** Pairwise comparison of compressive strength and Barcol hardness among the four nanohybrid composite resins. Statistical analysis was performed using Tukey's honest significant difference (HSD) post-hoc test following one-way analysis of variance. Data are presented as mean differences with 95% confidence intervals. Group I = Tetric N-Ceram, Group II = Prevest Fusion Universal, Group III = Dentgist NanoCom, Group IV = Waldent NanoFill. p < 0.05 was considered statistically significant; * indicates a statistically significant difference. CI = confidence interval; MPa = megapascal.

Intergroup comparison	Compressive strength				Barcol hardness number			
	Mean difference (MPa)	95% CI (Lower–Upper)	t-value	p-value	Mean difference	95% CI (Lower–Upper)	t-value	p-value
Group I vs. Group II	16.34	−13.42 to 46.10	1.61	0.463	7.60	4.80 to 10.40	11.31	< 0.001*
Group I vs. Group III	3.51	−23.45 to 30.47	0.36	0.979	17.60	14.80 to 20.40	30.40	< 0.001*
Group I vs. Group IV	23.42	−6.78 to 53.62	2.05	0.176	1.00	−1.80 to 3.80	1.524	0.768
Group II vs. Group III	-12.83	−42.59 to 16.93	–2.02	0.628	10.00	7.20 to 12.80	13.91	< 0.001*
Group II vs. Group IV	6.08	−27.08 to 39.24	0.80	0.950	-6.60	−9.40 to −3.80	–8.43	< 0.001*
Group III vs. Group IV	19.91	−10.29 to 50.11	2.43	0.282	-16.60	−19.40 to −13.80	–23.58	< 0.001*

## Discussion

The present in vitro study compared the physical and mechanical properties of four commercially available nanohybrid composite resins by evaluating their tensile strength, flexural strength, compressive strength, and Barcol hardness under standardized laboratory conditions. The findings demonstrated significant inter-material differences in the tensile strength, flexural strength, and surface hardness, whereas the compressive strength was comparable among the tested composites. Overall, Tetric N-Ceram exhibited the most favorable mechanical performance, particularly with respect to tensile strength, flexural strength, and hardness, indicating that not all nanohybrid composites possess equivalent mechanical characteristics, despite belonging to the same material category.

Tensile strength is an important predictor of the ability of a restorative material to resist crack initiation and propagation under functional stress. In the present study, Tetric N-Ceram demonstrated the highest mean tensile strength, while Waldent NanoFill exhibited the lowest values. The superior tensile strength of Tetric N-Ceram can be attributed to its optimized filler loading, improved filler-matrix coupling through silane treatment, and balanced resin matrix composition, which collectively enhance the stress distribution and reduce crack propagation [8]. In contrast, the lower tensile strength of some commercially available composites may reflect differences in the filler morphology, particle size distribution, resin composition, and polymerization efficiency. Similar observations were reported by Meenakumari et al. [7], who found that nanohybrid composites with a higher filler loading demonstrated superior tensile and fracture resistance. Likewise, Beun et al. [9] reported that the mechanical behavior of nanofilled and nanohybrid composites is highly dependent on the filler architecture rather than on the filler size alone.

Flexural strength represents the ability of a material to resist bending forces and is considered one of the most clinically relevant mechanical properties because restorations are frequently subjected to complex combinations of tensile, compressive, and shear stresses during mastication [10]. In the present study, Tetric N-Ceram demonstrated significantly greater flexural strength than DentGist NanoCom and Waldent NanoFill, while Fusion Universal also exhibited significantly higher flexural strength than these two materials. The observed differences indicate that the evaluated nanohybrid composites are not mechanically equivalent. Previous studies have shown that the flexural performance of resin composites is influenced by factors such as filler characteristics, resin matrix composition, polymer network integrity, and degree of monomer conversion. Ferracane [11] emphasized the importance of filler loading and polymer network integrity in determining flexural behavior, while Randolph et al. [12] reported that filler characteristics are major determinants of the physico-mechanical properties of resin composites. However, as the present study did not investigate the compositional characteristics or degree of monomer conversion of the tested materials, the precise mechanisms responsible for the observed differences cannot be established and warrant further investigation.

Although Tetric N-Ceram exhibited the highest mean compressive strength, no statistically significant differences were observed among the four materials. This finding suggests that all tested nanohybrid composites possess compressive strength values that are adequate to withstand the compressive occlusal forces encountered during routine mastication. Compressive strength is generally influenced by filler concentration, resin composition, and internal porosity; however, because modern nanohybrid composites are designed with a relatively high filler content, the differences among products may be less pronounced. Nagrale et al. [13] reported that nanocomposites exhibit significantly greater compressive and flexural strengths than conventional composites owing to their optimized filler particle size, higher filler loading, and improved filler-matrix interactions. These characteristics enhance the stress distribution within the resin matrix and improve resistance to functional loading. Similar findings were reported by Meenakumari et al. [8], who observed comparable compressive strengths among several contemporary posterior restorative composites despite differences in manufacturer formulations.

Surface hardness is considered an indirect indicator of wear resistance, filler content, and degree of polymerization. In the present study, highly significant differences in the Barcol hardness were observed among the tested materials. Tetric N-Ceram exhibited the highest hardness values, closely followed by Waldent NanoFill, whereas DentGist NanoCom demonstrated the lowest hardness. These findings suggest a considerable variation in the filler characteristics and polymer network formation among the evaluated composites. Greater hardness generally reflects increased filler loading, superior filler dispersion, and higher monomer conversion, all of which contribute to an improved resistance to surface wear and abrasion. Bala et al. [14] demonstrated that hardness values are strongly influenced by curing efficiency and polymerization characteristics, while Ernst et al. [15] reported that nanofilled composites exhibiting higher surface hardness showed superior clinical wear resistance during long-term follow-up.

The significantly higher Barcol hardness observed for Tetric N-Ceram in the present study indicates superior surface hardness compared with most of the other evaluated materials. Surface hardness is influenced by multiple factors, including filler characteristics, resin matrix composition, and the degree of polymerization. Sarma and Nagar [16] similarly reported that resin composites with differences in formulation exhibited corresponding differences in surface hardness. However, because the present study did not evaluate the compositional characteristics or degree of conversion of the tested materials, the exact factors responsible for the observed differences cannot be established and warrant further investigation.

The overall findings of the present study support the concept that commercially available nanohybrid composites cannot be considered to be mechanically equivalent. Although manufacturers frequently classify these materials under the same category, substantial differences exist in filler technology, particle morphology, resin chemistry, photoinitiator systems, and manufacturing protocols, resulting in variable mechanical performances. These observations are consistent with previous investigations by Willems et al. [17], Mitra et al. [18], and Elfakhri et al. [19], who concluded that filler characteristics are the principal determinants of composite mechanical behavior. Consequently, clinicians should consider independently generated evidence rather than relying solely on the manufacturers' claims when selecting restorative materials for stress-bearing clinical situations.

Clinical implications

The findings of the present study have important clinical implications in restorative dentistry. Materials demonstrating superior tensile strength, flexural strength, and surface hardness are expected to exhibit greater resistance to fracture, occlusal wear, and marginal deterioration, thereby contributing to the improved longevity of direct composite restorations, particularly in posterior stress-bearing regions. Based on the present findings, Tetric N-Ceram demonstrated the most favorable overall mechanical profile among the evaluated composites and may therefore be preferred in clinical situations where high functional loading is anticipated. Nevertheless, material selection should consider other clinically relevant factors, including polymerization shrinkage, esthetics, handling characteristics, marginal adaptation, and long-term clinical performance.

Limitations

The present study had certain limitations. Being an in vitro investigation, the present study could not reproduce the complex oral environment, including thermal fluctuations, cyclic occlusal loading, saliva, moisture, enzymatic degradation, and pH variations that influence the long-term performance of restorative materials. In addition, artificial aging procedures, such as thermocycling, prolonged water storage, and mechanical fatigue loading, were not performed. Therefore, the findings represent the baseline mechanical properties of the evaluated materials. Future studies incorporating artificial aging protocols and long-term clinical evaluation are warranted to better correlate laboratory findings with clinical performance. Only four mechanical properties were evaluated, whereas other clinically relevant parameters, such as wear resistance, fracture toughness, depth of cure, polymerization shrinkage, water sorption, color stability, and fatigue behavior, were not assessed. Furthermore, the relatively small number of commercially available composites evaluated limits the generalizability of our findings. Future studies should incorporate thermocycling, mechanical fatigue loading, aging protocols, and long-term clinical trials to correlate laboratory findings with clinical outcomes.

## Conclusions

Within the limitations of this in vitro study, commercially available nanohybrid composite resins exhibited significant differences in their mechanical properties. Tetric N-Ceram demonstrated the most favorable overall mechanical performance under the experimental conditions employed. These findings provide comparative laboratory evidence that may assist in material selection; however, they should not be interpreted as direct indicators of clinical performance. Long-term in vivo and well-designed clinical studies are required to confirm these findings and establish their clinical relevance.
